# Role of Exciton Diffusion in the Efficiency of Mn Dopant Emission in Two-Dimensional Perovskites

**DOI:** 10.1021/acsnanoscienceau.4c00047

**Published:** 2024-11-07

**Authors:** Alvaro J. Magdaleno, Anuraj S. Kshirsagar, Marc Meléndez, Udara M. Kuruppu, Jesse J. Suurmond, Mercy M. Cutler, Michel Frising, Michael Seitz, Rafael Delgado-Buscalioni, Mahesh K. Gangishetty, Ferry Prins

**Affiliations:** 1 Condensed Matter Physics Center (IFIMAC), Autonomous University of Madrid, Madrid 28049, Spain; 2 Department of Condensed Matter Physics, Autonomous University of Madrid, Madrid 28049, Spain; 3 Department of Chemistry, Mississippi State University, Mississippi State, Mississippi 39762, United States; 4 Department of Theoretical Condensed Matter Physics, Autonomous University of Madrid, Madrid 28049, Spain

**Keywords:** 2D perovskites, Mn doping, exciton diffusion, transient microscopy, carrier dynamics

## Abstract

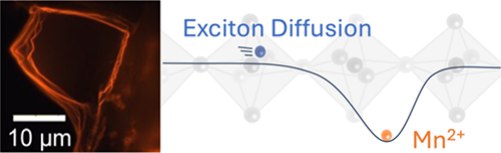

Two-dimensional (2D) metal-halide perovskites have promising characteristics for optoelectronic applications. By incorporating Mn^2+^ ions into the perovskite structure, improved photoluminescence quantum yield can be achieved. This has been attributed to the formation of defect states that act as efficient recombination centers. Here, we make use of transient photoluminescence microscopy to characterize important material parameters of Mn^2+^-doped 2D perovskites with different doping levels. From these measurements, we visualize the importance of exciton transport as an intermediate step in the excitation of Mn^2+^. We model the spatiotemporal dynamics of the excited states to extract the diffusion constant and the transfer rate of the excitations to the Mn dopant sites. Interestingly, from these models, we find that the average distance an exciton needs to travel before transferring to a Mn site is significantly larger than expected from the Mn concentration obtained from elemental analysis. These insights are critical from a device design perspective.

## 1 Introduction

2D metal-halide perovskites have emerged as a versatile class of semiconductors for light-emitting applications. Owing to their layered structure, they exhibit strong quantum confinement and high exciton binding energies with high photoluminescence quantum yields (PLQYs). Their emission is broadly tunable through both compositional tuning and thickness variations.^1−6^ Consequently, they are explored heavily in light-emitting devices (LEDs) and photovoltaic (PV) applications.^7^ Particularly, PEA_2_PbBr_4_ (where PEA = phenethylammonium) are the most studied materials in blue and sky-blue LEDs due to their superior color stability, unlike their mixed Cl/Br halide 3D perovskites counterparts.^7−10^ Recently, doped strategies have been demonstrated further to control emission quantum yields, colors, and stability.^7,11−19^ Many metal ions, including mono (Li^+^ and K^+^), bi (Mn^2+^, Ni^2+^, Cu^2+^, and Zn^2+^), and trivalent metals (Bi^3+^, Y^3+^, Er^3+^, and Yb^3+^), have been used as dopant ions in 2D perovskites.^20,21^ While some metal ion dopants exhibit their characteristic emission peaks, others improve the perovskites’ optical and electronic properties, including their stability.^22^

Mn^2+^ ions are among the most popular dopant ions. Mn^2+^-doping imposes fascinating exciton-dopant interactions that yield bright orange emission (with the band edge emission) at ∼600 nm with high PLQY. In addition to the dopant emission, Mn^2+^ doping has been shown to improve the emission quantum yield of its 2D perovskite hosts. By doping with Mn^2+^, up to near unity PLQYs have been achieved in blue and green perovskites.^13,14,17^ As a result, Mn^2+^-doped perovskites are employed in LEDs,^12,23−25^ lasers,^26^ spintronics,^27,28^ and as X-ray scintillators in imaging applications.^29^ Recently, LEDs made from Mn-doped perovskites have been reported with superior performance and stability across the blue, green, and red spectra compared to undoped ones.^12,23−25^ By carefully tuning the Mn doping level in blue perovskites, one can attain a desirable mixing of blue and orange emissions to achieve white light emission from single components.^30,31^ The bright orange emission from the Mn state is attributed to the ^4^T_1_-to-^6^A_1_ (*d*-*d*) transition of the Mn^2+^ dopant. These states are long-lived with a lifetime of several microseconds and are typically populated through the excitation of the host. The Mn population relies on the efficiency of energy transfer from the host perovskite lattice to the Mn^2+^ states. This transfer is driven by short-range exchange interactions between the *d* electrons of the Mn and the *sp* spins of the excitonic energy carriers of the host perovskite.^13,32−34^ Despite the short-range exchange interactions, efficiencies in Mn^2+^-doped systems are remarkably high. It is important to note in this regard that excitons in two-dimensional metal-halide perovskites are highly mobile^35−37^ and diffusion-mediated transfer of energy likely plays an important role in the high efficiency of Mn^2+^-doped perovskite emission.

Here, we elaborate on the specific role of exciton diffusion in the emissive properties of Mn^2+^-doped perovskites. We study the case of single crystalline two-dimensional (PEA)_2_PbBr_4_ perovskite, grown by acid precipitation and slow evaporation. By using different concentrations of Mn^2+^ precursor during the synthesis, we obtain a family of layered perovskites with distinct dopant levels. We show that the dopant level has a significant influence on exciton diffusion within the extended plane of the perovskite layers using transient microscopy measurements.^35,38,39^ Using diffusion theory, we extract the trapping rate at Mn sites for different doping levels and visualize the deep trap nature of the Mn defects.

## 2 Results

**Figure 1:**
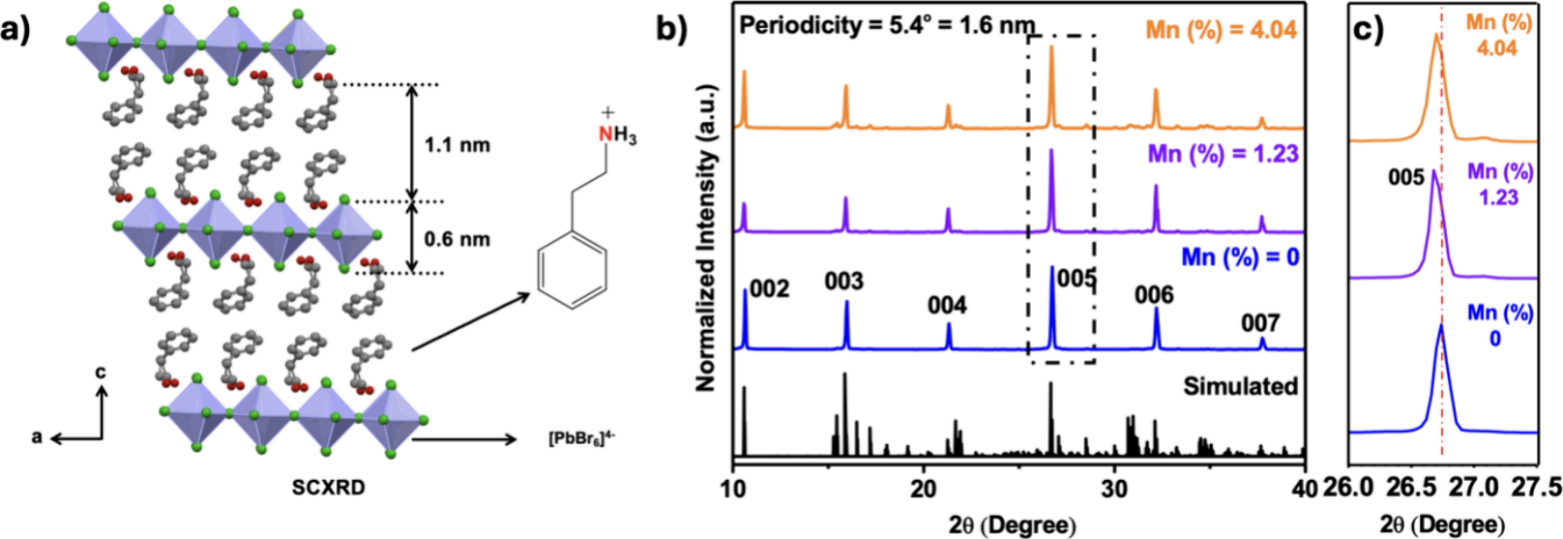
(a) (PEA)_2_PbBr_4_ crystal structure showing the Ruddlesden–Popper phase with an interlayer spacing of 1.7 nm. (b) Powder XRD of undoped and Mn^2+^-doped perovskites (1.23 and 4.04%) and the corresponding simulated pattern is shown in the bottom row. (c) Expanded region of the (005) plane, showing the shift in diffraction peak with Mn^2+^ doping.

Mn^2+^-doped (PEA)_2_PbBr_4_ perovskites are synthesized by acid-initiated precipitation (see the Experimental Section for more details), resulting in high-purity 2D perovskite crystals. The crystal structure of undoped perovskite crystals derived from scXRD is shown in Figure 1a, which confirms the presence of Ruddlesden–Popper phases with an interlayer spacing of 1.7 nm. The refined data is shown in Table S1. This value agrees well with the spacing (1.6 nm) derived from the periodicity (5.4°) of XRD peaks and the literature data (Figure 1b).^40^ Upon doping with Mn^2+^, we observed no significant changes in their periodicity, except a slight shift in the XRD peaks. Figure 1c shows the peak at ∼26° indexed to the (005) plane, shifting to lower angles, indicating a subtle change in the lattice parameters after doping with Mn^2+^ ions. The shift to the lower angles suggests a lattice expansion along the (005) planes (*c*-axis). Generally, according to the Goldsmith tolerance factor, when a small metal ion, such as Mn^2+^, replaces/substitutes Pb^2+^ in the lattice, the resultant lattice undergoes contraction, and the XRD peaks shift to higher angles. On the contrary, the peak shift to lower angles after Mn^2+^ doping implies that there is a lattice expansion along the *c*-axis due to the Mn^2+^ ions occupying an interstitial position instead of replacing Pb, as suggested by Torma et al.^41^

**Figure 2:**
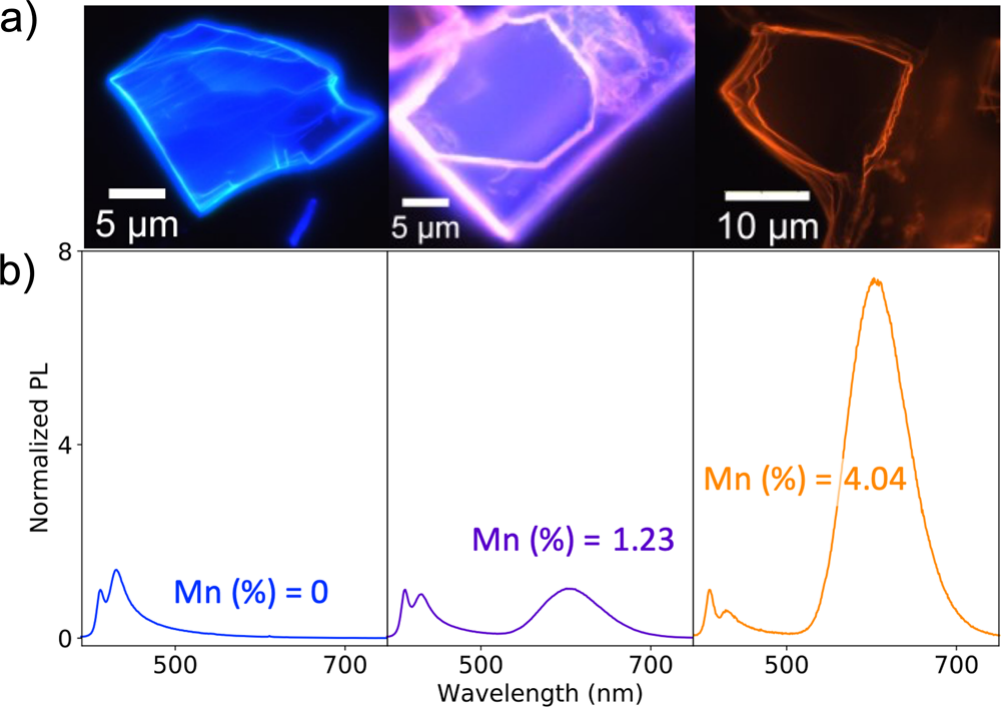
(a) Exfoliated flakes from the synthesized Mn^2+^:(PEA)_2_PbBr_4_ crystals with 0, 1.23, and 4.04% doping. (b) Photoluminescence emission spectrum for each doping, an increased orange emission is observed for the higher doping levels. Spectra are normalized to the intensity at 390 nm.

The presence of Mn^2+^ ions was confirmed by ICP-MS analysis, and the corresponding Mn(%) denoted here refers to the Mn^2+^:Pb^2+^ molar ratio in the perovskite lattice. Further, the flakes are isolated by mechanical exfoliation of the high-purity crystals and transferred to a microscope slide. Figure 2a shows fluorescence microscopy images of three different doping levels (0, 1.23, and 4.04%), clearly demonstrating the variability of the color of emission due to doping. The emission spectra in Figure 2b demonstrate how the increasing orange emission intensities with increasing doping levels of the Mn^2+^ ions are responsible for the overall color change. The Mn^2+^ emission peak is centered at 590 nm and has a relatively large full-width-at-half-maximum of 277 meV, corresponding to an octahedral environment with high crystal field strength and suggesting either [MnBr_6_]^4–^ or [MnBr_4_(H_2_O)_2_]^4–^ coordination.^42^

**Figure 3:**
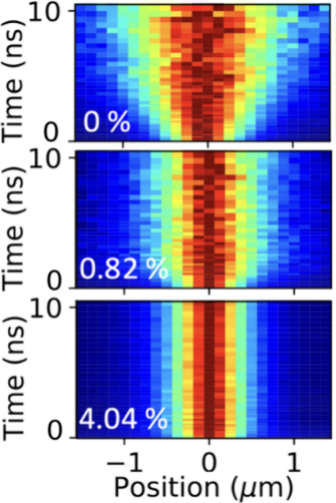
Spatial broadening of the exciton population over time for 0, 0.82, and 4.04% doped perovskites. The intensity profiles at each time-slice are normalized to emphasize the broadening of the populations, which is inhibited for higher doping levels. Diffusion maps are recorded without spectral selection.

To verify the role of exciton diffusion in the excited state dynamics of Mn-doped (PEA)_2_PbBr_4_, we perform transient photoluminescence microscopy (TPLM) on individual exfoliated flakes of the material. The basic operating mechanism of this technique is illustrated and explained in previous reports.^35,43,44^ In short, we place a scanning avalanche photodiode (20 μm in size) in the magnified (330 × ) image plane of the emission profile of the exciton population. Using pulsed laser excitation (*<*200 ps, 50 nJ cm^–2^, 40 MHz), photoluminescence lifetime traces are recorded for different positions along the emission profile, allowing for a reconstruction of the in-plane spatiotemporal evolution of the exciton population with subnanosecond precision. In Figure 3, we show the spatial and temporal evolutions for different doping levels (0, 0.82, and 4.04%). Importantly, an inhibition of the population expansion is observed when increasing the Mn-doping, consistent with trapping of the excitons at Mn sites.

**Figure 4:**
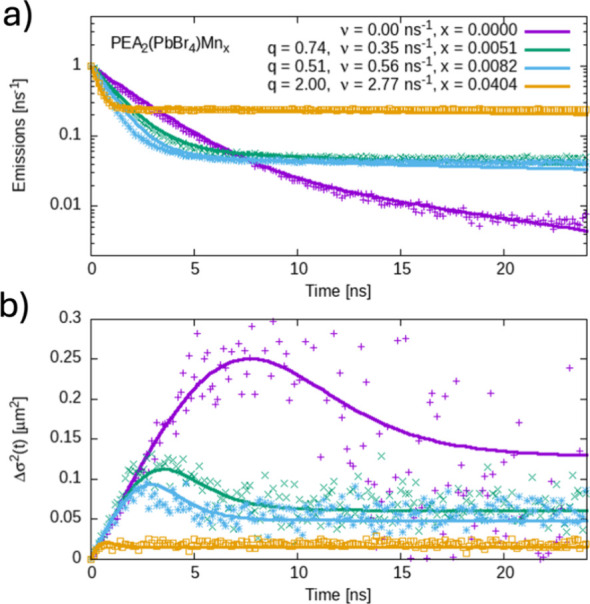
(a) Population decay for 0 (purple), 0.51 (green), 0.82 (blue), and 4.04% (orange) Mn^2+^-doped perovskites. (b) Change in observed variance Δσ^*2*^(*t*) for the same set of samples. Solid lines in (a) and (b) indicate global fits where, except for the values indicated, all other parameters were kept constant across samples. See the SI, Section S3, for the full list of parameters.

The broadening of the spatial distribution of PL emission can be quantified by tracking the increase in variance Δσ^*2*^(*t*) = σ^*2*^(*t*) – σ^*2*^(0). The variance σ^2^(*t*) is obtained from fitting a Voigt function to the intensity profile for each time slice.^35,43^ In Figure 4, we show the population decay (top panel) and the change in observed variance Δσ^2^(*t*) (bottom panel) for 0, 0.51, 0.82, and 4.04% doping levels. The doped samples display a rapid initial decay corresponding to the depopulation of the perovskite band edge, followed by a slower decay corresponding to the decay from the Mn dopant sites. As expected, the depopulation of the band edge excitons becomes faster with increasing doping levels, indicating an increase in the trapping rate.

For the change in observed variance Δσ^2^(*t*), a common initial linear regime is observed, which is associated with the transport of the free excitons within the perovskite host lattice.^43^ This linear regime is shortened when increasing the dopant concentrations, favoring earlier subdiffusive regimes where the excitons get trapped (and decelerated) as they reach the Mn^2+^-states. The deceleration of the expansion is followed by an apparent contraction of the carrier population, indicative of a short-lived fast-diffusing species and a long-lived trapped species.^45,46^ It is important to mention the observed dynamics of the undoped perovskite, where notable deviations from normal diffusion are already present (e.g., nonmonoexponential decay and nonlinear diffusion at later times). We attribute these dynamics to the presence of intrinsic traps, as we will discuss in more detail later.

Using a diffusion model, we can understand the observed dynamics in the different samples and explore the nature of the Mn-traps. Several different rates are involved in the process: the trapping rate (ν), the detrapping rate (μ), the decay rate of free excitons (γ_free_), and the decay rate of trapped excitons at Mn sites (γ_Mn_). Assuming that excitons cannot escape deep traps (μ = 0), we obtain the following rate equation for the populations of both the free excitons *N*_free_ and the trapped excitons *N*_Mn_:
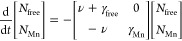
1

In addition, we suppose that direct excitation of Mn states is negligible (*N*_free_(0) ≫ *N*_Mn_(0)), so we can set *N*_free_(0) = 1 and *N*_Mn_(0) = 0. This yields the following expressions for the exciton populations:
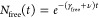
2and
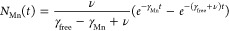
3

In our model, freely diffusing excitons spread out with a variance proportional to time. In the dilute regime, a diffusing exciton does not interact with others, and the trapping rate remains independent of the local concentration of excitons. If *D*_0_ stands for the diffusivity of free excitons, then
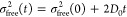
4

At every small time-interval of length d*t* a fraction of the free population equal to *N*_free_ν d*t* and variance σ_free_^2^(*t*) becomes trapped. Note that the positions of particles trapped at time *t* are not correlated with the positions of other particles trapped previously. Therefore, the variance of the trapped population becomes the following weighted average:
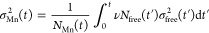
5where we made use of the simplifying assumption that, since γ_Mn_ ≪ γ_free_, trapped populations hardly have time to decay significantly over the time interval in which most of the excitons fall into traps or disappear (a more rigorous derivation confirms the validity of the approximation when γ_Mn_ ≪ γ_fre*e*_, see below and the SI). Carrying out the integral,
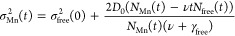
6which predicts the following height for the plateau at later times:
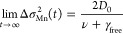
7

Importantly, because free and trapped excitons emit photons at different rates and because they are detected with different probabilities (due to the spectral dependence of the quantum efficiency of the APD), each population contributes to the variance observed in experiments proportionally to the number of emitted photons.
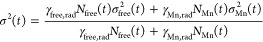
8

The expression above includes only the radiative decays, equal to the decay rates seen previously times the quantum yield for each state. Thus, for example, γ_free,rad_ = *q*_free_γ_free_*y*_free_, where *q*_free_ stands for the quantum yield for free excitons and *y*_free_ for their probability of detection taking into account the quantum efficiency of the detector.

Finally, because of the long lifetime of the Mn^2+^ emission, we must consider the laser pulse repetition rate. The small value of γ_Mn_ leads to a situation that for sufficiently rapid pulsing, when a laser pulse arrives, a significant number of trapped excitons created by previous pulses is still in the excited state. This lingering population can be included alongside that of the most recent pulse in the calculation of both populations and variances. For the number of excitons, this extra background term amounts to an additional population decaying at the rate γ_Mn_, with the initial population determined by summing up contributions from the train of previous laser pulses. If *t*_p_ stands for the time between consecutive laser pulses, then
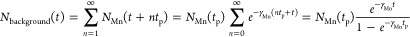
9

Regarding the influence of the laser repetition on the variance, we can consider that the background contributes with an additional term equal to the long-time variance 2*D*_0_/(γ_free_ + ν), weighted by the background emission level.

Using this formalism, we can fit the time and spatially resolved exciton dynamics of the different doping levels. For this, we first turn to the undoped perovskite as a benchmark for the general behavior. Importantly, despite the absence of Mn^2+^ dopants, a transition to a subdiffusive regime is nevertheless observed at later times and this is accompanied by a nonmonoexponential population decay. This suggests the presence of intrinsic traps in these materials, an observation which is consistent with earlier reports on 2D perovskites.^35,45^ To account for this, we include a small population of additional deep traps that are intrinsic to the undoped perovskite as part of our fit procedure (see SI, Section S3 for details), constant in all samples. Solid lines in Figure 4a,b represent the fits obtained from our model experimental data for lifetimes and Δσ^*2*^(*t*). In this global fit, the same parameter values are used for both lifetime decay and Δσ^*2*^(*t*) for each material. Importantly, apart from the trapping rate of excitons into the Mn^2+^ traps (*k*_trap_) and the quantum yield for excitons in the Mn^2+^ state (*q*_Mn_), all other parameters remain constant across different samples (see Table S2 for the parameter values).

## 3 Discussion

**Figure 5:**
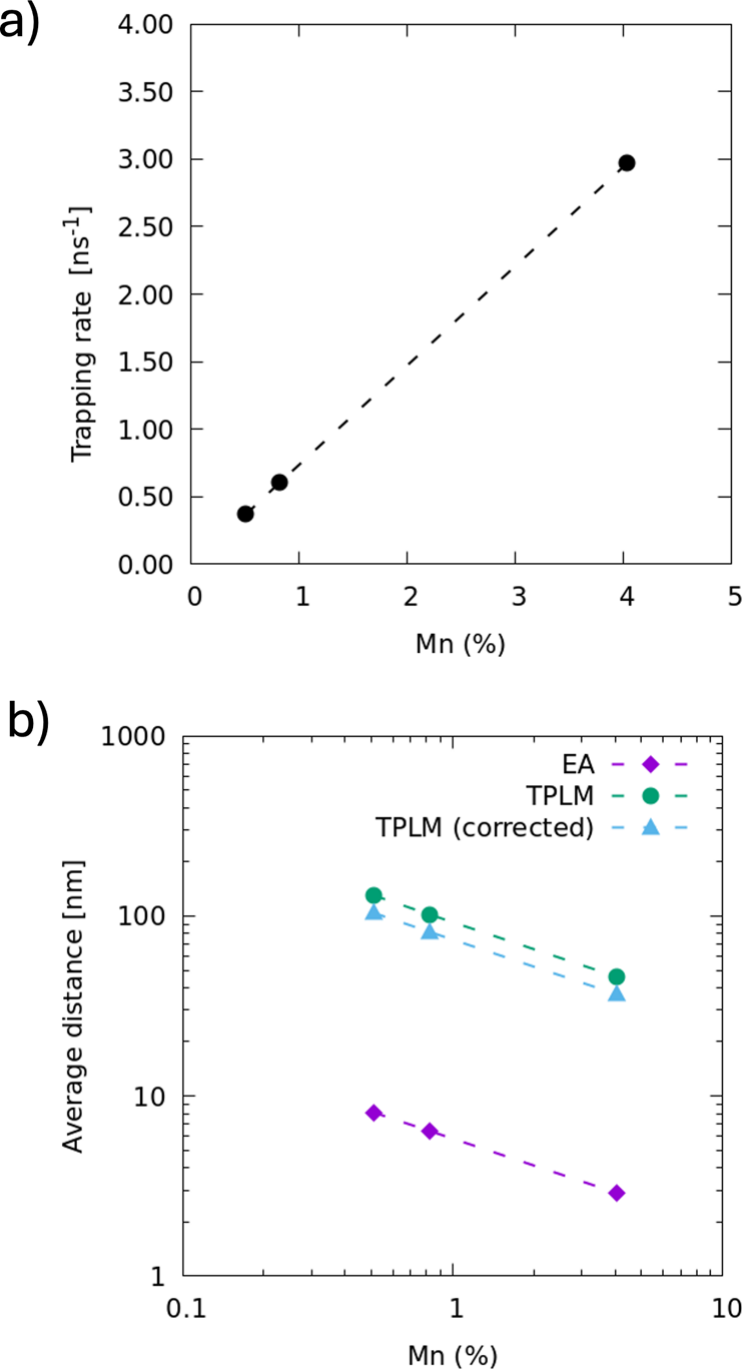
(a) Trapping rates for the different doping levels obtained from the fits in Figure 4. (b) Average distance between the closest Mn sites as estimated by elemental analysis (purple diamonds), fits to transient photoluminescence microscopy experiments using the formalism presented in the main text (green circles), and fits to transient photoluminescence microscopy experiments in which nonlinear effects related to trap-state filling have been taken into account (blue triangles). Dashed lines are guide to the eye.

The global fits of the spatiotemporal data allow us to obtain a full description of the exciton dynamics governing the Mn^2+^ dopant emission in 2D perovskites and emphasize the critical role of the diffusion of excitons through the two-dimensional plane in the transfer efficiency of energy from the perovskite to the Mn^2+^ dopants. The average distance between Mn sites δ_average_ can be estimated using the lattice constants of the perovskite *a*. Assuming a random distribution of the dopants within the lattice, δ_average_ ∼ *a*/(2*C*_Mn_^1/2^), with *C*_Mn_ the Mn/Pb fraction. From the fits we obtain both the trapping rate ν and the diffusion constant of the excitons *D*_0_ = 0.25 cm^2^/s, allowing us to determine the time needed for the excitons to reach a trap by simple diffusion, δ_average_^2^/*D*_0_. If the trapping events were diffusion limited, this time would coincide with the inverse of the trapping rate, τ_d_ = 1/ν. However, as Figure 5 indicates, we find (1/ν) ∼ 2 × 10^3^τ_d_, which suggests that the Mn^2+^ sites are not homogeneously distributed or that the transition from free to trapped exciton is slowed down by an additional barrier for energy transfer to the Mn^2+^ dopants.

One possible explanation for the apparent slow trapping rate would be that the exciton-polaron limits the interaction with the Mn^2+^ sites, in a similar way as the polaron formation screens the excitons from scattering and defect states. Using Kramer’s reaction time (with trial-time τ_*d*_), such a barrier can be estimated to be of order *k*_B_*T* ln(τ_d_ν) ∼ 150 meV. This is, however, considerably larger than the typical values associated with the stabilizing energy of exciton-polarons in 2D metal-halide perovskites (30–50 meV),^47^ meaning polaron formation in itself is not be able to explain the slow trapping rate. A reduced trapping rate could alternatively originate from the physical separation between the excitons in the inorganic lattice of the perovskites and the interstitial Mn^2+^ dopant. An additional energy transfer rate of 0.1 ps^–1^ would explain the discrepancy for the different doping levels (see the SI, Section S5 for more details).

An alternative explanation for the discrepancy between ν and τ_*d*_ comes from possible nonrandom distributions of Mn^2+^ ions in the perovskite lattice. As shown in Figure 4, the diffusion length explored in 1/ν time is 10-times larger than δ for randomly placed Mn^2+^ sites. Hence, Mn^2+^ rich regions with *N*_*c*_ ∼ 100 sites would be required to explain the observed delayed trapping, i.e., (*D*_0_/ν)^1/2^ ∼ 10 δ_average_. Finally, our diffusion equation formalism does not include nonlinear effects such as trap-state filling. Indeed, a saturation of the Mn^2+^ emission intensity can be observed for increasing excitation power indicating the filling of dopant sites (see the SI, Section S4). While our measurements are taken relatively far from saturation, some trap state filling is indeed already present. Taking such nonlinearities into account in our model, we find that this only accounts for a small reduction in the effective distance between Mn^2+^ sites (see blue triangles in Figure 5 and a detailed description of the extended model in the SI, Section S4).

## 4 Conclusions

In conclusion, we have directly visualized the role of exciton diffusion in the Mn^2+^ emission of Mn^2+^-doped 2D-perovskites, demonstrating that exciton transport is inhibited as the Mn^2+^-doping level is increased. By comparing the full spatiotemporal excited state dynamics of Mn:(PEA)_2_PbBr_4_ perovskite crystals with different doping levels, we have developed a detailed model that includes the deep trap nature of the Mn^2+^-states and allows us to extract the average distances between Mn-states. These distances are significantly larger than what would be expected from elemental analysis. This discrepancy can be either due to nonhomogeneous distributions of Mn^2+^ sites or related to the protective nature of exciton-polaron formation in 2D perovskites. Further studies will be needed to elucidate this point. Our work provides critical insights into the design of optimally efficient Mn^2+^-doped 2D perovskites, highlighting the dominant role of exciton diffusion in the population of the Mn^2+^ sites. It suggests that Mn^2+^-doping strategies will be most efficient in perovskite lattices with higher diffusion constants.

## 5 Experimental Section

### 5.1 Materials

Lead bromide (PbBr_2_, 99.999%), phenethylammonium bromide (C_8_H_12_BrN, 98%), manganese(II) bromide (MnBr_2_, 98%), hydrobromic acid (HBr, 48%), and nitric acid (HNO_3_, ≥99.999% trace metals basis) were purchased from Sigma-Aldrich and used as received. PEA_2_PbBr_4_ single crystals were synthesized by an acid-initiated precipitation method.^1^ In this method, 305 mg (0.833 mmol) of PbBr_2_, 335 mg (1.66 mmol) of C_8_H_12_BrN, and various amounts of MnBr_2_ (to maintain Mn^2+^: Pb^2+^precursor mole ratios of 1:0.2, 1:0.5, 1:1, and 4:1) were dissolved in 10 mL of HBr. The precursor solution was heated to 120 °C under constant magnetic stirring to obtain a clear and transparent solution. Stirring was then stopped, and the solution was allowed to cool to room temperature at a controlled cooling rate of 0.5 °C/min. Finally, the precipitated crystals were suction-filtered and dried under reduced pressure. Powder X-ray diffraction (PXRD) was performed using a PROTO AXRD benchtop powder diffraction system with Cu K_α_ radiation (1.54 Å). See Table S1 for the scXRD refined parameters. Steady-state photoluminescence (PL) spectra were recorded using an Edinburgh FS5 spectrofluorometer. For ICP-MS analysis, exactly 10.0 mg of the sample was digested in HNO_3_. Then the ICP-MS analysis was carried out (PerkinElmer ELAN DRC II ICP-MS) and the Mn^2+^ mole percentage was calculated with respect to the Pb^2+^ moles.

### 5.2 Transient Photoluminescence Microscopy

Mechanically exfoliated single crystals of Mn:(PEA)_2_PbBr_4_ perovskites were prepared using the scotch tape (Scotch Magic) method. After several exfoliation steps, crystals were transferred on a glass slide, yielding flakes of around 100 nm thick and several tens of microns in size. Transient Photoluminescence Microscopy (TPLM) was subsequently performed by imaging through the glass slide with a × 100 oil immersion objective (Nikon CFI Plan Fluor, NA = 1.3) onto the center of the perovskite flakes. TPLM was performed as previously described.^35,39,45^ In short, a near-diffraction-limited exciton population was generated using a 405 nm pulsed laser diode (PicoQuant LDH-D-C-405, PDL 800-D; 40 MHz, 50 nJ cm^–2^). The emission from the exciton population was collected using a optically magnified 330 times using a Nikon CFI Plan Fluor objective (NA = 1.3) and subsequently imaged with a scanning avalanche photodiode (APD, Micro Photon Devices PDM, 20 × 20 μm^2^ detector). The laser diode and APD were synchronized using a Pico-Harp 300 timing board for time-correlated single-photon counting. During measurements, the sample was scanned over an area of 5 × 5 μm^2^ with an x-y piezo stage (MCL Nano-BIOS 100) to minimize photodegradation of the perovskite flakes. The time binning of the measurement setup was set to 4 ps before software binning was applied. We follow the same fitting procedure as described previously by our group to extract the evolution of the variance Δσ^*2*^(t) of the exciton population.^35^

## Supporting Information Available

Additional material characterization, details of transient microscopy measurements and data analysis (PDF)
