# Supplementary material for: Role of Exciton Diffusion in the Efficiency of Mn Dopant Emission in Two-Dimensional Perovskites
Source: ACS Nanosci Au. 2024 Nov 7;5(1):29–36. doi: 10.1021/acsnanoscienceau.4c00047 (PMC11843506; doi:10.1021/acsnanoscienceau.4c00047)
Supplement: Supplementary file 1 — ng4c00047_si_001.pdf [file ng4c00047_si_001.pdf]

The role of exciton diffusion in the efficiency of Mn-dopant emission in 2D perovskites

Alvaro J. Magdaleno,[1,24] Anuraj S. Kshirsagar,[3] Marc Meléndez,[1,] Udara M. Kuruppu,[3] Jesse J. Suurmond,[1,2] Mercy M. Cutler,[1,2] Michel Frising,[1,2] Michael Seitz,[1,2] Rafael Delgado-Buscalioni,[1,4] Mahesh K. Gangishetty,[3] and Ferry Prins[1,2]**

[1]Condensed Matter Physics Center (IFIMAC), Autonomous University of Madrid, 28049, Madrid, Spain

[2]Department of Condensed Matter Physics, Autonomous University of Madrid, 28049, Madrid, Spain

[3]Department of Chemistry, Mississippi State University, Mississippi State, Mississippi 39762, United States

[4]Department of Theoretical Condensed Matter Physics, Autonomous University of Madrid, 28049, Madrid, Spain

S1. scXRD Refined Parameters

**Chemical formula**	$C_{32}H_{48}Br_8N_4Pb_2$	
**Formula weight**	1542.40 g/mol	
**Temperature**	100(2) K	
**Wavelength**	0.71073 Å	
**Crystal size**	(0.091 x 0.102 x 0.234) mm^3	
**Crystal system**	triclinic	
**Space group**	P -1	
**Unit cell dimensions**	a = 11.5219(6) Å	α = 80.385(2)°
	b = 11.5254(6) Å	β = 73.921(2)°
	c = 17.2743(10) Å	γ = 89.966(2)°
**Volume**	2170.7(2) $Å^3$	
**Z**	2	
**Density (calculated)**	2.360 g/cm^3	
**Absorption coefficient**	15.136 mm^{-1}	
**F(000)**	1424	
**Theta range for data collection**	1.91 to 30.50°	
**Index ranges**	-16<=h<=16, -16<=k<=16, -24<=l<=24	
**Reflections collected**	72441	
**Independent reflections**	13232 [R(int) = 0.0605]	
**Coverage of independent reflections**	99.7%	
**Absorption correction**	Multi-Scan	
**Max. and min. transmission**	0.3400 and 0.1260	
**Refinement method**	Full-matrix least-squares on F^2	
**Refinement program**	SHELXL-2018/3 (Sheldrick, 2018)	
**Function minimized**	Σ w(F_o^2 - F_c^2)^2	
**Data / restraints / parameters**	13232 / 0 / 458	

**Goodness-of-fit on F^2**	1.063	
**Δ/σ_{max}**	0.001	
**Final R indices**	11289 data; I>2σ(I)	R1 = 0.0345<br>wR2 = 0.0927
	all data	R1 = 0.0427<br>wR2 = 0.0984
**Weighting scheme**	$w=1/[\sigma^2(F_o^2)+(0.0531P)^2+3.9105P]$<br>where $P=(F_o^2+2F_c^2)/3$	
**Extinction coefficient**	0.0014(1)	
**Largest diff. peak and hole**	2.625 and -3.117 eÅ^{-3}	
**R.M.S. deviation from mean**	0.306 eÅ^{-3}	

Table S1: scXRD Refined Parameters

S2. Analytical reaction-diffusion theory for excitons in perovskite layers

Suppose that $c_i(r,t)$ represents the concentration of excitons in state i (with i = free, Mn, 2) at position $\boldsymbol{r}$ and time t, and that these concentrations obey the following set of reaction-diffusion equations.

$$\frac{\partial c_{\text{free}}}{\partial t} = D_0 \nabla^2 c_{\text{free}} - (\nu + \nu_2 + \gamma_{\text{free}}) c_{\text{free}},$$

$$\frac{\partial c_{\text{Mn}}}{\partial t} = \nu c_{\text{free}} - \gamma_{\text{Mn}} c_{\text{Mn}},$$

$$\frac{\partial c_2}{\partial t} = \nu_2 c_{free} - \gamma_2 c_{2.}$$

Integrating these equations over space and using the fact that

$$\int D_0 \, \nabla^2 c_{\text{free}} dr = 0,$$

produces equations for the total populations.

$$\frac{\partial N_{\text{free}}}{\partial t} = -(\nu + \nu_2 + \gamma_{\text{free}}) N_{\text{free}},$$

$$\frac{\partial N_{\text{Mn}}}{\partial t} = \nu N_{\text{free}} - \gamma_{\text{Mn}} N_{\text{Mn}},$$

$$\frac{\partial c_2}{\partial t} = \nu_2 N_{\text{free}} - \gamma_2 N_{2.}$$

Similarly, if we define the quantities

$$V_i(t) = \int r^2\, c_i(r,t) dr,$$

multiply the reaction-diffusion equations by r^2 and integrate over space, then we obtain the following equations,

$$\frac{\partial V_{\text{free}}}{\partial t} + (\nu + \nu_2 + \gamma_{\text{free}})V_{\text{free}} = 4D_0 N_{\text{free}}.$$
$$\frac{\partial V_{\text{Mn}}}{\partial t} - \nu V_{\text{free}} + \gamma_{\text{Mn}} V_{\text{Mn}} = 0.$$
$$\frac{\partial V_2}{\partial t} - \nu_2 V_{\text{free}} + \gamma_2 V_2 = 0.$$

From these functions, we can calculate the variance of the populations in each state as

$$\sigma_i^2(t) = \frac{V_i(t)}{N_i(t)}.$$

Solving the differential equations for the$N_i$andV_i, we obtain the following analytical expressions for the populations and variances.

$$N_{\text{free}}(t) = N_{\text{free}}(0) e^{-(\nu+\nu_2+\gamma_{\text{free}})t},$$
$$N_{\text{Mn}}(t) = \frac{\nu N_{\text{free}}(0)}{\nu + \nu_2 + \gamma_{\text{free}} - \gamma_{\text{Mn}}} \left(e^{-\gamma_{\text{Mn}}t} - e^{-(\nu+\nu_2+\gamma_{\text{free}})t}\right),$$
$$N_2(t) = \frac{\nu_2 N_{free}(0)}{\nu + \nu_2 + \gamma_{free} - \gamma_2} \left(e^{-\gamma_2 t} - e^{-(\nu+\nu_2+\gamma_{free})t}\right),$$
$$\sigma_{\text{free}}^2(t) = 2D_0 t,$$
$$\sigma_{Mn}^2(t) = 2D_0 N_{Mn}(t) \left(\frac{1}{\nu + \nu_2 + \gamma_{free} - \gamma_{Mn}} - \frac{t}{e^{(\nu+\nu_2+\gamma_{free}-\gamma_{Mn})t} - 1}\right),$$
$$\sigma_2^2(t) = 2D_0 N_2(t) \left(\frac{1}{\nu + \nu_2 + \gamma_{\text{free}} - \gamma_2} - \frac{t}{e^{(\nu+\nu_2+\gamma_{\text{free}}-\gamma_2)t} - 1}\right).$$

As emphasized in the main text, the actual populations $N_{i,\text{total}}$and variances $\sigma_{i,\text{total}}^2$at time *t* arise as a superposition of the effects of a train of pulses separated by times t_p.

$$N_{i,\text{total}}(t, t_p) = \sum_{n=0}^{\infty} N_i\left(t + n t_p\right).$$

However, we can approximate the background variance by means of the long-time plateau and assume that no free excitons remain from previous pulses.

$$\sigma_{i,\text{total}}^2(t, t_p) = \frac{N_i(t)\sigma_i^2(t) + N_{i,\text{background}}(t)\left(\lim_{t\to\infty} \sigma_i^2(t)\right)}{N_{i,\text{total}}(t)}.$$

The transient PL experiments register light emitted from excitons in every state, which means that the total emission should be calculated as

$$E(t) = \sum q_i\, \gamma_i y_i N_{i,\text{total}}(t),$$

where $q_i\gamma_i$ stands for the radiative emission rate for excitons in state i and y_i for the probability of detection of the resulting photon. Concerning the observed variance, the same type of reasoning leads to

$$\sigma_{\text{total}}^2(t) = \frac{\sum q_i\, \gamma_i y_i \sigma_{i,\text{total}}^2(t)}{E(t)}.$$

It is the emissions $E(t)$ and observed variance $\sigma_{\text{total}}^2(t)$ that we compare directly to experimental measurements.

S3. Simultaneous fitting of the lifetime and variances introducing an extra trap state

As mentioned in the main text, the long-time dynamics of the sample without any Mn doping reveal the presence of a deep trap state which modifies the slope of the decay and contracts the observed variances (Fig. 4), which is why we considered an additional type of trap with subindex 2. Fitting the undoped curve to the approximate model for a single type of trap as presented in the main text provides an estimate of the corresponding trapping and decay rates ($\nu_2 = 0.02\ \text{ns}^{-1}$, $\gamma_2 = 0.1\ \text{ns}^{-1}$). We neglect detrapping and non-radiative decay for these additional traps. The diagram in Fig S1 summarizes all the relevant decay processes considered.

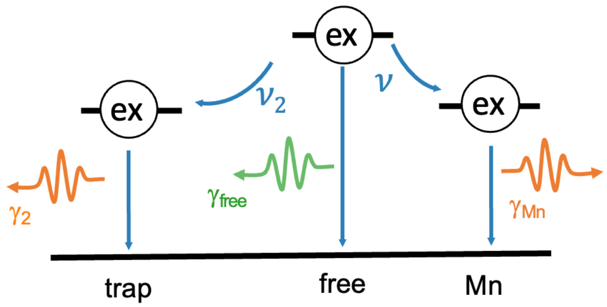

Figure S1: Diagram of the decay processes involved in the model of exciton diffusion that includes an additional type of trap even for undoped samples.

The parameters for the additional traps were determined from fitting the approximate model from the main text to the undoped sample. The following parameters were fixed for all the lifetime and $\Delta\sigma^2$ curves.

- $t_p = 25$ ns time between pulses.
- $D_0 = 0.025\,\mu\,\mathrm{m}^2/\mathrm{ns}$ diffusivity of free excitons.
- $\gamma_{\mathrm{free}} = 0.47\ \mathrm{ns}^{-1}$ decay rate for free excitons.
- $\gamma_{\mathrm{Mn}} = 0.008\ \mathrm{ns}^{-1}$ decay rate for excitons in Mn traps.
- $\nu_2 = 0.02\ \mathrm{ns}^{-1}$ trapping rate for additional traps.
- $\gamma_2 = 0.1\ \mathrm{ns}^{-1}$ decay rate for additional traps.
- $y_{\mathrm{free}} = 0.25$ probability of detection for photons from free exciton recombination.
- $y_{\mathrm{Mn}} = 0.37$ probability of detection for photons from exciton recombination in Mn^{2+} states.
- $q_2 = 1.00$ quantum yield of additional traps.

The remaining parameter values appear in Table S2.

Sample [% Mn]	$q = q_{\mathrm{Mn}}/q_{\mathrm{free}}$	$\nu[\ \mathrm{ns}^{-1}]$
0%	n/a	0.000
50%	0.74	0.35
80%	0.51	0.56
400%	2.00	2.77

Table S1: Variable fit parameters for Fig. 4: $q = q_{Mn}/q_{\mathrm{free}}$, the ratio of quantum yield for excitons in Mn traps to quantum yield for free excitons, and ν, rate for exciton trapping in states created by Mn impurities.

S4. Variation of the trapping rate with laser fluence

A very rough order-of-magnitude estimation of the variation of the trapping rate ν with laser fluence could begin by assuming that the initial population of excitons generated by the incident light pulse lies in direct proportion to the laser fluence. The fraction of this population that gets trapped in Mn states and then emits light would be proportional to the trapping rate. Consequently, we can postulate that the trapping rate varies approximately linearly with the number of photons emitted by Mn states divided by the laser fluence. The same conclusion could be derived from the equation for N_{Mn} above, by noting that $N_{Mn}(t) < N_{free}(0)$,$\gamma_{Mn} \ll \nu + \nu_2 + \gamma_{free}$, $\gamma_{Mn} \ll 1$, and remembering that $N_{free}(0)$ equals the fluence multiplied by some constant. This approximation neglects the contribution of filled Mn^{2+} states from previous laser pulses, but we will show below that a more rigorous derivation leads to the same conclusion.

Due to trap state filling, however, we expect the effective trapping rate to decrease as the fluence increases. Note that the trapping rate is proportional to the concentration c_{Mn} of Mn sites, which,

if arranged randomly on the plane, are separated by a distance $d \approx 0.5(c_{Mn})^{-1/2}$. This means that when the effective trapping rate changes by a factor of α, then apparent distance changes by a factor of $\alpha^{-1/2}$.

Fig. S2 displays the integrated intensities of light detected within the range of frequencies corresponding to the radiative decay of Mn^{2+} states, together with the how the emission-to-fluence fraction falls off with increasing fluence. For fluences of 0.1 μW (which correspond to the data presented in this work) the trend implies a fall in the trapping rate of about 30–40%, meaning that the actual distance between closest Mn sites would be about 20% smaller than estimated from the trapping rates extracted from fits to the TPLM data in the main text. This estimation clearly points out that disregarding trap state filling can only account for a modest fraction of the difference observed between TPLM and elemental analysis estimations of the concentration of Mn sites. Most of the disagreement should be attributed to other causes, such as clustering or energy barriers, as discussed in the main text.

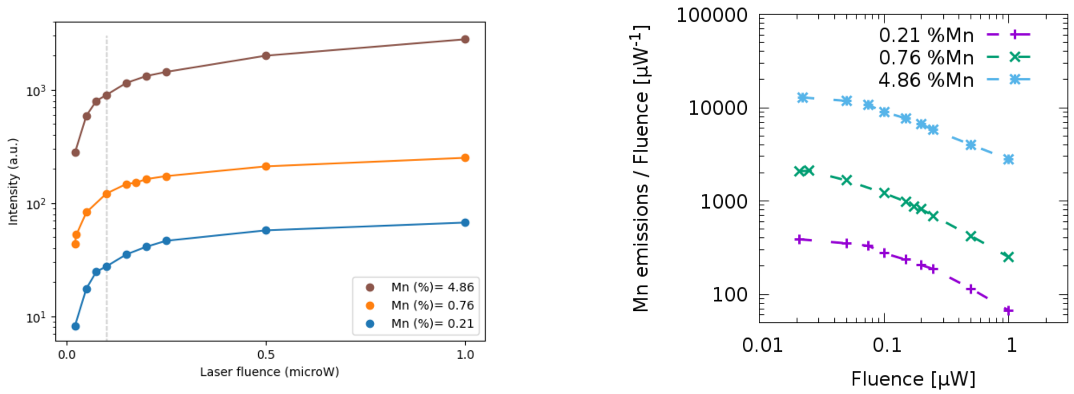

Figure S2: *Left*: integrated intensity of light emitted in the range corresponding to radiative decay from Mn^{2+} sites versus laser fluence. *Right*: Mn^{2+} emission to fluence ratio (proportional to trapping rate) versus fluence.

We now show that a more careful deduction of the relation between total emission and laser fluence agrees with the back-of-the-envelope estimation derived above. The total time-integrated measured intensity equals

$$I = \gamma_{Mn} q_{Mn} \int_0^{t_p} N_{Mn,total}(t) dt = \int_0^{\infty} N_{Mn}(t) dt.$$

Carrying out the integration,

$$I = \gamma_{Mn} q_{Mn} \frac{\nu N_{free}(0)}{\nu + \nu_2 + \gamma_{free} - \gamma_{Mn}} \left(\frac{1}{\gamma_{Mn}} - \frac{1}{\nu + \nu_2 + \gamma_{free}} \right).$$

If we use the approximation $\gamma_{Mn} \ll \nu + \nu_2 + \gamma_{free}$, then the expression becomes

$$I \approx q_{Mn}\frac{\nu N_{free}(0)}{\nu + \nu_2 + \gamma_{free} - \gamma_{Mn}}.$$

Solving for the trapping rate,

$$\nu \approx \frac{(\nu_2 + \gamma_{free})I}{q_{Mn}N_{free}(0) - I} \approx \frac{(\nu_2 + \gamma_{free})I}{q_{Mn}N_{free}(0)},$$

where we have used the fact that in our experiments, $q_{Mn}N_{free}(0) \gg I$, because the number of detected photons from Mn^{2+} states I must be smaller than $q_{Mn}\gamma_{Mn}\nu N_{free}(0)$, and $\gamma_{Mn}\nu \ll 1$. Assuming that the number of excitons created lies in direct proportion to the fluence of the laser pulse, we arrive at the conclusion stated above that the trapping rate will be roughly proportional to the Mn^{2+} emission to fluence ratio.

S5. Estimation of additional energy transfer rate

If we hypothesize that Mn^{2+} dopants lie at interstitial sites, then we can explain the difference between the trapping rate in the models and that estimated from elemental analysis as the result of an additional energy transfer rate. This would mean that trapping does not occur immediately when the exciton reaches a trapping site, but that there is some probabilistic rate of energy transfer from the perovskite layer to the interstitial Mn^{2+}. We find that the probability of an exciton diffusing to a trap (estimated from elemental analysis) equals about 250 times the probability of getting trapped. This amounts to a conditional probability factor of $p = 0.004$ of getting trapped after finding a trap. Supposing that energy transfer becomes possible when the exciton lies within a distance of $r = 1$nm, then the time spent near a trap equals $\pi r^2/(4D_0)$, and the rate of energy transfer equals $4D_0\, p/(\pi r^2) = 127$ ns^{-1}.

References

(1) Seitz, M.; Meléndez, M.; Alcázar-Cano, N.; Congreve, D. N.; Delgado-Buscalioni, R.; Prins, F. Mapping the Trap-State Landscape in 2D Metal-Halide Perovskites Using Transient Photoluminescence Microscopy. *Adv. Opt. Mater.* **2021**, *2021*, 2001875. https://doi.org/10.1002/adom.202001875.
